# Perniosis-like erythema after SARS-CoV2 infection reactivated by vaccination with inactivated virus^⋆^

**DOI:** 10.1016/j.abd.2022.01.015

**Published:** 2023-03-30

**Authors:** Lucas Campos Garcia, Vanessa Martins Barcelos, Myrciara Macedo de Alcântara, Priscila Jordana Costa Valadares

**Affiliations:** a Department of Dermatology, Hospital das Clínicas, Universidade Federal de Minas Gerais, Belo Horizonte, MG, Brazil; b Dermatology Private Practice, Curitiba, PR, Brazil

Dear Editor,

Chilblain-like or perniosis-like erythema (PLE) has been frequently reported in association with SARS-CoV2 infection.^1^ With the advancement of vaccination against this virus, there have also been reports of these lesions after the use of messenger RNA (mRNA) vaccines.^2^ This report describes a case in which such lesions manifested after the infection and recurred with the patient receiving a vaccine against COVID-19 containing inactivated virus.

A 71-year-old female patient presented with acrocyanosis and erythematous infiltrated papules on the fingers in June 2020. One week before, she had presented with asymptomatic SARS-CoV2 infection, confirmed by RT-PCR (real time – polymerase chain reaction) testing. The condition was treated with dapsone 50 mg/day, with improvement after two months of use. The patient had a previous history of central nervous system vasculitis, diagnosed seven years before, based on neurological manifestations – without cutaneous symptoms, under remission for six years using azathioprine 2 mg/kg/day.

In May 2021, the lesions recurred one week after the first dose of the CoronaVac vaccine and persisted for two months, when dermatological care was introduced (Fig. 1). She had no systemic symptoms. Histopathology of a skin biopsy showed vacuolar degeneration of the basal layer, apoptotic keratinocytes, superficial and deep perivascular and periadnexal lymphohistiocytic inflammatory infiltrate, in addition to endothelial edema of small vessels (Fig. 2), findings that can be observed in drug reactions and viral infections. Laboratory tests for collagenosis were negative. SARS-CoV2 reinfection was ruled out by RT-PCR.

**Figure 1:**
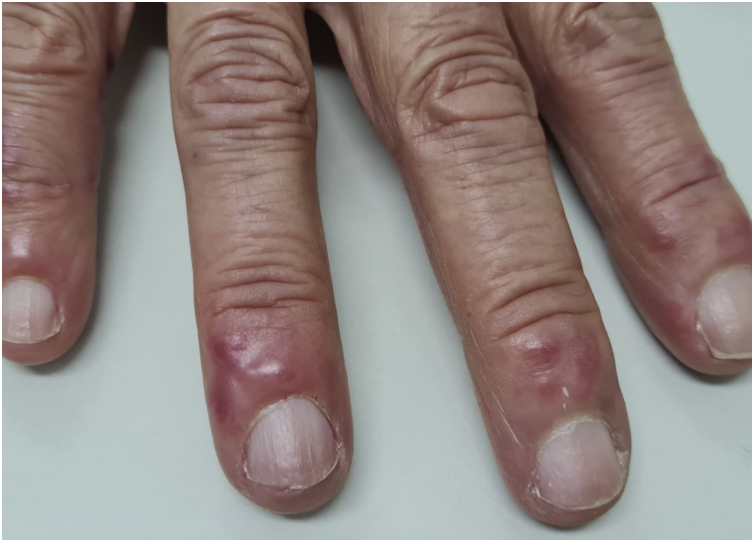
Mild acrocyanosis and erythematous-infiltrated papules on the distal region of the fingers.

**Figure 2:**
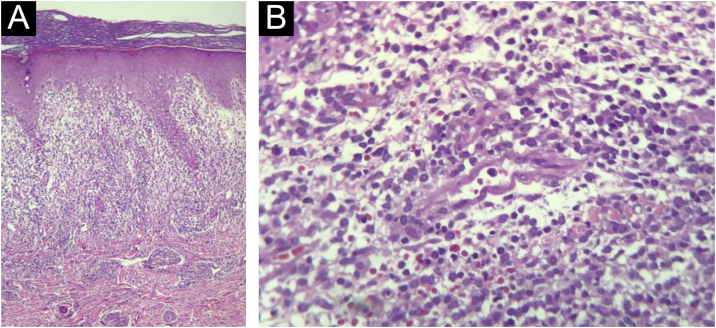
(A) Elongation of epidermal rete pegs and lymphocytic inflammatory infiltrate in the dermoepidermal interface and upper papillary/reticular dermis. Vacuolar degeneration of the basal layer and apoptotic keratinocytes can be observed, in addition to extravasated red blood cells (Hematoxylin & eosin, ×100). (B) In the dermis, a small-caliber vessel with swollen endothelium, demonstrating vascular aggression. Surrounding it, lymphohistiocytic inflammatory infiltrate and extravasation of red blood cells can be observed (Hematoxylin & eosin, ×400).

Hence, the diagnosis of PLE after SARS-CoV2 infection, which was reactivated by the vaccine with the inactivated virus was raised. Due to the absence of systemic symptoms and criteria for vasculitis, the hypothesis of central nervous system vasculitis reactivation was refuted. Dapsone 100 mg/day and amlodipine 5 mg/day were prescribed, the latter to manage the acrocyanosis. After one month of drug use, there was improvement in the inflammatory reaction, and persistence of acrocyanosis (Fig. 3).

**Figure 3:**
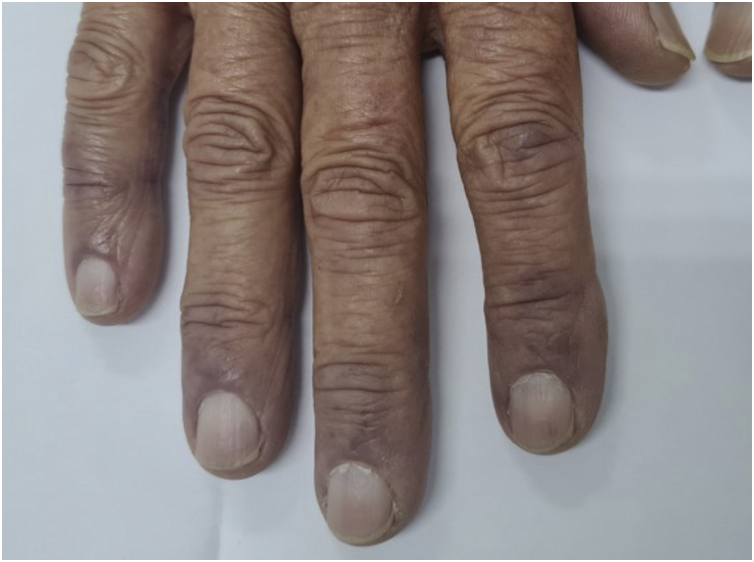
Persistence of acrocyanosis and full resolution of papules.

Classic erythema pernio usually occurs after exposure to cold, damp weather. It may be associated with acrocyanosis and autoimmune diseases, such as systemic lupus erythematosus. However, the frequency of this association is still controversial.^3^

SARS-CoV2-associated PLE usually occurs after the second week of infection and is associated with mild or asymptomatic disease in young, healthy patients.^1^ There have also been reports of occurrence after immunization against COVID-19, mostly with mRNA vaccines.^2^ To the best of the authors’ knowledge, only two cases of PLE have been previously reported following vaccination with the inactivated SARS-CoV2 virus vaccine, i.e., the CoronaVac.^4^ However, the present case is the first in which post-infection PLE (confirmed by RT-PCR) recurred after the administration of the vaccine with the inactivated virus.

The authors believe that their observation supports the hypothesis that vaccines induce an infection-like immune reaction driven by type I interferon.^5^ The recurrence of PLE lesions after an inactivated virus vaccine also suggests these manifestations are related to the immune reaction to the virus, and not directly to the virus.

Given the importance of immunization advances to limit the current pandemic, the authors’ objective with this report is to provide information for the future understanding of adverse reactions and post-vaccination advice.

## Financial support

None declared.

## Authors' contributions

Lucas Campos Garcia: Approval of the final version of the manuscript; design and planning of the study; drafting and editing of the manuscript; intellectual participation in the propaedeutic and/or therapeutic conduct of the studied cases; critical review of the literature; critical review of the manuscript.

Vanessa Martins Barcelos: Drafting and editing of the manuscript; critical review of the literature; critical review of the manuscript.

Myrciara Macedo de Alcântara: Drafting and editing of the manuscript; critical review of the literature; critical review of the manuscript.

Priscila Jordana Costa Valadares: Drafting and editing of the manuscript; critical review of the literature; critical review of the manuscript.

## Conflicts of interest

None declared.
